# Perinatal MAO Inhibition Produces Long-Lasting Impairment of Serotonin Function in Offspring

**DOI:** 10.3390/brainsci8060106

**Published:** 2018-06-11

**Authors:** Mark W. Burke, Myriam Fillion, Jose Mejia, Frank R. Ervin, Roberta M. Palmour

**Affiliations:** 1 Department of Physiology and Biophysics, College of Medicine, Howard University, Washington, DC 20059, USA; mark.burke@Howard.edu; 2 Departments of Biology, McGill University, Montréal, QC H3A 1A1, Canada; myriam.fillion@teluq.ca; 3 Department of Psychiatry, Dalhousie University, Halifax, NS B3J 3T4, Canada; jose.mejia@iwk.nshealth.ca; 4 Department of Psychiatry, McGill University, Montréal, QC H3A 1A1, Canada; rmpskb@gmail.com; 5 Human Genetics, McGill University, Montréal, QC H3A 1A1, Canada

**Keywords:** serotonin transporter, dopamine transporter, neurodevelopment, MAO inhibition, aggression, anxiety

## Abstract

In addition to transmitter functions, many neuroamines have trophic or ontogenetic regulatory effects important to both normal and disordered brain development. In previous work (Mejia et al., 2002), we showed that pharmacologically inhibiting monoamine oxidase (MAO) activity during murine gestation increases the prevalence of behaviors thought to reflect impulsivity and aggression. The goal of the present study was to determine the extent to which this treatment influences dopamine and serotonin innervation of murine cortical and subcortical areas, as measured by regional density of dopamine (DAT) and serotonin transporters (SERT). We measured DAT and SERT densities at 3 developmental times (PND 14, 35 and 90) following inhibition of MAO A, or MAO B or both throughout murine gestation and early post-natal development. DAT binding was unaltered within the nigrostriatal pathway, but concurrent inhibition of MAO-A and MAO-B significantly and specifically reduced SERT binding by 10–25% in both the frontal cortex and raphe nuclei. Low levels of SERT binding persisted (PND 35, 90) after the termination (PND 21) of exposure to MAO inhibitors and was most marked in brain structures germane to the previously described behavioral changes. The relatively modest level of enzyme inhibition (25–40%) required to produce these effects mandates care in the use of any compound which might inhibit MAO activity during gestation.

## 1. Introduction

Low levels of serotonin and its metabolite 5-hydroxyindoleacetic acid (5-HIAA) are thought to be associated with aggression and impulsivity [1,2,3,4,5,6,7,8]. A striking example of this relationship is a single Dutch kindred [9,10] in which approximately half of the male offspring displayed mild mental retardation and aggressive impulsive behavior linked to a point mutation of the monoamine MAO-A gene [10]. MAO-A is one of two isoforms of MAO which selectively oxidizes serotonin, norepinephrine and epinephrine, the other being MAO-B which preferentially oxidizes phenylethylamine with both isoforms having the ability to oxidize dopamine [11]. Inhibition of MAO has been successfully used as an antidepressant in adults and is not known to cause aggressive impulsive behavior [12]. In affected individuals of the Dutch kindred, MAO-A activity was ablated, and levels of serotonin and serotonin metabolites were abnormally high, thus providing a paradox with respect to the “low serotonin-elevated aggression” model [10,13]. 

Mice genetically engineered to be deficient in MAO-A and/or MAO-B activity [14,15,16,17,18] reinforce the deterministic nature of this mutation. Male MAO-A knockout mice exhibit enhanced context-related aggression together with increases in monoamines during development [14,19,20] possibly reflecting an inherent inability to appropriately assess social cues. Anatomically, MAO-A knockout mice have reduced levels of the serotonin transporter (SERT) in the dorsal raphe [17], as well as higher somatosensory and lower entorhinal cortical activation as compared to wildtype controls [21]. These anatomical alterations are accompanied with modified gene expression associated with neurodevelopment, apoptosis, and cognitive function which may be related to the behavioral and anatomical abnormalities reported with altered serotonin levels during development [22]. By contrast, MAO-B knockout mice display neither aberrant behavior nor detectable changes in monoamines, with the exception of increased levels of phenylethylamine [23]. MAO-A/B double knockout mice have even higher levels of serotonin than MAO-A knockouts, and also have increased levels of dopamine and norepinephrine. Accompanying the neurochemical changes, these mice exhibit abnormal fear conditioning and alterations in hippocampal long-term potentiation [18]. Similar to the MAO-A knockout mice, MAO-A/B knockout mice display increased aggression and anxiety-like behaviors and have been proposed as a model of autism spectrum disorder [15,16,22,24].

Perinatal partial inhibition of MAO-A/B by clorgyline (MAO-A selective) and deprenyl (MAO-B selective) also induces pervasive aberrant behavior in both mice and rats [25,26]. Perinatal inhibition of MAO in rats increased impulsivity-related behaviors in the passive avoidance paradigm, aggressive behavior toward cage-mates and handlers, impaired visual function, and reduced cortical serotonin innervation [25]. Perinatal inhibition of MAO-A/B in mice results in elevated aggressive behavior and a tendency toward impulsive responding, which extends beyond the point of MAO inhibition [26]. 

Deprivation of MAO during development provides a reliable and reproducible model in which to investigate pervasive aggressive and impulsive behaviors [25,26], but the absence of these behaviors in persons treated post-pubertally with MAO inhibitors [27] suggests that a direct causal relationship between MAO inhibition and aggressive behavior is unlikely. An alternative explanation would be that perinatal MAO inhibition has complex effects on the developing neural systems [28,29] as developmental expression and activity of MAO-A precedes that of MAO-B and therefore inhibition could differentially affect neural networks [30,31]. This study aims to investigate relative dopamine and serotonin innervation of cortical and subcortical areas following perinatal murine MAO inhibition (A, B or A/B). 

## 2. Materials and Methods

### 2.1. Subjects

CD1 timed-pregnant mice (*n* = 4/group) were purchased from Charles River Laboratory and were received one day after impregnation. After one day of acclimation, the animals were weighed and minipumps were prepared for each animal. The following day, under ketamine/xylazine anaesthesia the pregnant dams were implanted with ALZET osmotic minipumps (model 1002; two-week pump) filled with clorgyline (MAO-A inhibited group), l-deprenyl (MAO-B inhibited group), a combination of clorgyline and l-deprenyl (MAO-AB inhibited group), or 0.9% sterile saline (control group). The delivery rate for each pump was 0.25 µL/hour so that each dam would receive 0.25 mg/kg/day deprenyl, 1 mg/kg/day clorgyline, or the two drugs in combination. The animals were subjected to MAO inhibition in utero and then to a lower level of MAO inhibition due to the presence of the inhibitors in the dams’ milk. In order to maintain MAO inhibition for a total of 6 weeks (3 weeks of foetal development and 3 weeks of post-natal development) the minipumps had to be replaced. To prevent interference with the mother-infant bonding period, the initial pumps were surgically replaced on E17, two days before parturition (ALZA model 2004; four-week pump). Male pups from each treatment group were randomly assigned to the post-natal (PND) 14, 35, and 90-day groups. With respect to postnatal exposure to MAO inhibitors (MAO-I), the PND 14 group still had active inhibition (through the mother’s milk) at the time of sacrifice, but this terminated at PND 21. 

All studies were done in accordance with the guidelines of the Canadian Council of Animal Care, and were reviewed and approved by the University Animal Care Committee of McGill University.

### 2.2. Behavioral Assessment

Neurological Assessment: Neurological evaluations were performed as previously described [26]. Briefly, a random sample of the pups (*n* = 51) from both the PND 35 or PND 90 time points, were evaluated between 30 and 40 post-natal days on 14 tests according to standard procedures [32] in order to identify early signs of neurological impairment. Briefly, subjects were observed for 1. Pivoting behavior (circular locomotion caused by side motion of the front legs with hind legs essentially inactive), 2. Straight walking (locomotion in an approximate straight line with all four legs involved), 3. Righting reflex (turning to rest in a normal position on all four legs after being placed on the side by the experimenter), 4. Back righting (turning to rest in a normal position on all four legs after being placed on the back the by the experimenter), 5. Rooting reflex (pushing forward after bilateral stimulation of the face area), 6. Cliff drop aversion (withdrawing from the edge of a surface when the forepaws are placed over the edge), 7. Grab reflex (foot flexion with grasping of a metal rod when the plantar surface is stroked by the rod), 8. Front placing (raising and placing foot on the table top when dorsum of foot is placed in contact on the edge of the table top while the animal is suspended by loose skin from the back of the neck during testing), 9. Crossed extensor (flexion of hind limb when pinched and extension of opposite hind limb), 10. Bar holding (grasping a wooden pencil with the front legs and paws and supporting their own weight), 11. Auditory startle (immediate flight or “freezing reaction” following hand clap of experimenter), 12. Eyes open (post-natal time of eye opening), 13. Hyperreactivity (overreaction to novel stimuli, i.e., the “popcorn stage” where the animal displays exaggerated freezing or jumping elicited by external noise and/or movements), and 14. Mass reaction (exaggerated squirming and rolling with occasional whole body convulsion following tail pinch). Behaviors were assessed on a 3–4 point scale that ranged from absent of behavior to full response. Total time for neurological tests was less than 30 min for each subject.

Behavioural Tests: Mouse pups assigned to the PND 90 time point (*n* = 6/treatment group) were behaviourally tested between 30 and 40 days of post-natal life in an Omnitech animal activity monitor between 8:30 a.m. and 11:30 a.m. for three minutes in order to determine total locomotor activity levels, time spent in the centre and margins of the open field and to detect aberrant locomotor behaviour such as excessive stereotypies. 

### 2.3. Brain Preparation

Mouse pups were sacrificed at 14, 35, or 90 days of age (*n* = 6/treatment group/time point) by cervical dislocation followed by decapitation. The brains were then quickly removed and frozen at −80 °C in 2-methylbutane. Serial sagittal sections (20 µm) were used to identify levels of DAT and serotonin transporter SERT autoradiographically.

### 2.4. Autoradiography

Sagittal sections (20 µm) were thaw mounted on gelatin-coated slides and stored at −80 °C. Quantification of DAT binding sites was performed by incubating sections in a sub-saturating concentration of ^125^I-RTI-55, a high affinity analogue of cocaine, according to standard protocols [33,34]. Briefly, sections were thawed and then incubated for two hours at room temperature with 10 pM ^125^I-RTI-55 (NEN/Perkin-Elmer) diluted in a buffer containing 10 mM sodium phosphate, 120 mM sodium chloride, 0.1 M sucrose, and 50 nM citalopram (pH 7.4). Citalopram was used to mask SERT during both incubation and washing. The sections were washed 3 times in buffer at 4 °C, dipped in double deionized water to remove buffer salts, dried and exposed to BioMax MS film for 3 days along with a ^125^I radioactive standard. Non-specific binding was defined as residual ^125^I-RTI-55 bound in the presence of 10 µM GBR12909.

Quantification of SERT binding sites was performed under the same conditions used for DAT, with a few notable exceptions. DAT was occluded with 1 µM GBR12935 in the incubation and wash buffer rather than citalopram. The concentration of labelled ligand (10 pM ^125^I-RTI-55) remained the same. Non-specific binding was defined as residual ^125^I-RTI-55 bound in the presence of 100 nM citalopram [34,35].

### 2.5. Image Analysis 

Quantification of the autoradiographic studies was performed using the public domain NIH Image program (developed at the U.S. National Institute of Health and available on the internet at http://rsb.info.nih.gov/nih-image/). Optical density readings from autoradiographs were transformed into fmol/mg bound using ^125^I microscale standards (Amersham), which were exposed on each film. 

### 2.6. Data Analysis 

Unless specified otherwise, data were evaluated with a two-way (treatment × time) analysis of variance, performed using StatView on a Macintosh computer. Significant findings (*p* < 0.05) were followed by Fisher’s post-hoc (PSLD) test.

## 3. Results

*Neurological test*: Scores obtained on the neurological tests confirm previous results (Mejia et al., 2002) in that neurological status did not differ (F_3,47_ = 1.2, *p* = 0.32) between the four groups.

*Open field test*: Locomotor activity in the open field test was not significantly changed by treatment (F_3,20_ = 1.12, *p* = 0.366). Stereotypy was also unaffected by treatment (F_3,20_ = 1.55, *p* = 0.23). Exploratory patterns, measured as time spent in the center of the enclosure as compared to time spent exploring the margins (F_3,20_ = 0.68, *p* = 0.57), were also not altered by MAO exposure (Table 1). 

*Histological Analysis*: In control subjects (as well as experimental) DAT and SERT densities increased over time in all measured areas in agreement with other developmental studies [36,37,38,39]. Table 2 shows that SERT and DAT typically reached adult levels by 35 days of age in most brain areas. In some brain stem regions (substantia nigra: DAT; raphe area: SERT), however, adult levels were reached by 14 PND (F_3,59_ = 0.94, *p* > 0.05 and F_2,51_ = 2.96, *p* > 0.05 respectively). 

**Figure 1:**
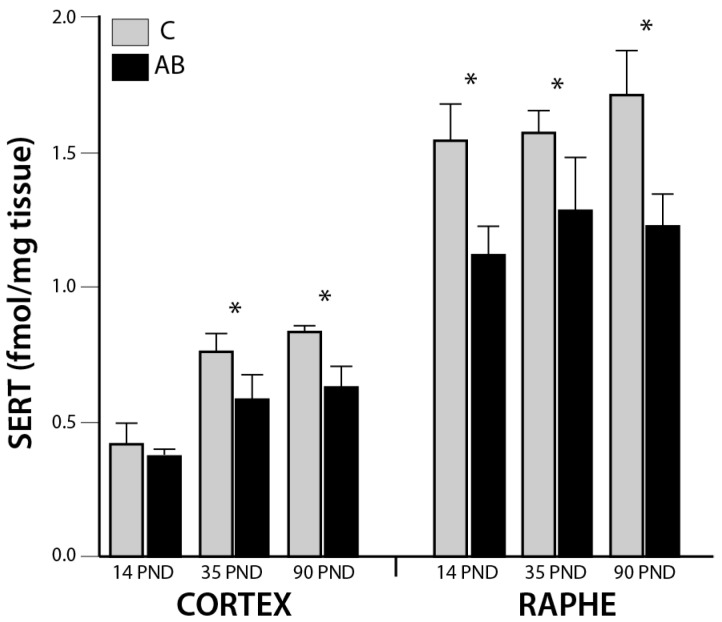
SERT Binding Densities. MAO inhibition with combined clorgyline and deprenyl (AB), as described in detail in the text, reduced SERT binding in the cortex (F_3,52_ = 3.59, *p* = 0.02) and the raphe nuclei (F_3,51_ = 6.07, *p* = 0.0015) in both a time- and treatment-dependent fashion. Significant pairwise time x treatment effects (*p* < 0.05) are indicated by *. In the cortex, AB was significantly different from all other groups (post-hoc *p* < 0.05); in raphe, AB was significantly different from C and A, and B was also significantly different from C (post-hoc *p* < 0.05). Six animals were examined for each treatment and each time point; error bars S.E.M.

In addition to the main effect of age, there was also a main effect of MAO inhibition on SERT binding in the raphe (F_3,51_ = 6.07, *p* = 0.0015; Figure 1). In this structure, post-hoc analysis showed significant pairwise differences between groups (AB versus C (*p* < 0.0001), B versus C (*p* < 0.01), and AB versus A (*p* < 0.01)). Reduced raphe SERT binding of group AB is apparent at each developmental time point (−27.4%, −18.8% and −27.7%, as compared to control, at PND 14, 35 and 90, respectively. Cortical reductions of SERT was also apparent after MAO inhibition (F_3,52_ = 3.59, *p* = 0.02; Figure 1) with significant pairwise differences between groups AB versus C (*p* = 0.0004), AB versus B (*p* < 0.05), and AB versus A (*p* < 0.02). For this region, the reduction relative to control (9.5% at PND 14, 19.2% at PND 35 and 26.2% at PND 90) appeared to be progressive (Table 3). There were no significant effects of treatment on SERT density in the other structures examined (hippocampus, striatum, substantia nigra) and no interaction of time × treatment in any region.

The effects of MAO inhibition on DAT expression during early development was much less marked than those reported for SERT. Two-way ANOVA failed to detect significant differences in DAT densities between groups (Table 3) in the striatum (F_3,61_ = 0.76, *p* = 0.52), nucleus accumbens (F_3,52_ = 0.35, *p* = 0.79), medial forebrain bundle (F_3,48_ = 0.78, *p* = 0.51) or substantia nigra (F_3,59_ = 0.94, *p* = 0.43). 

## 4. Discussion

MAO inhibitors are often used in the treatment of atypical depression and anxiety disorders, and even though they alter the levels of neurotransmitters in the brain [40,41], they have not been reported to increase aggressive behavior in adults [27]. By contrast, alterations in endogenous levels of neurotransmitters during the prenatal developmental period may have very different consequences [42]. Clinically, gestational exposure to selective serotonin reuptake inhibitors (SSRIs) is associated with adolescent diagnosis of depression and speech disorders [43,44] with a potential for an elevated risk of autism spectrum disorder diagnosis [45,46,47] although this remains controversial. A key issue is that, during development, serotonin most particularly has trophic effects that influence the ontogenesis of neurons and the organization of the nervous system [29,48,49,50,51,52]. This influence may occur either locally or through the maternal-placental-fetal forebrain pathway [53]. Levels of neural serotonin, in turn, are strongly influenced by the level of activity of the degradative enzyme MAO, which in turn is under complex developmental regulation [54]. 

Previous research from this laboratory shows that combined inhibition of MAO-A and B activity during murine development results in behaviors interpreted as aggressive and impulsive [26]. Selective inhibition of either MAO-A or MAO-B produced lower intensity behavioral alterations. In the present study, we evaluated the effect of these treatments on transporters of dopamine and serotonin, as these proteins have a major role in controlling available levels of the neurotransmitters. Consistent with the hypothesized effect, combined MAO-A/B inhibition significantly and specifically reduced SERT binding in the cortex and raphe nucleus throughout development and into adulthood, even when MAO inhibitors were no longer being administered (PNDs 35 and 90). Persistent effects were restricted to cortex and raphe nuclei, suggesting a relative vulnerability of these regions to early insult. Prenatal inhibition of MAO did not significantly affect DAT binding, although there was a transient effect (*p* < 0.05) toward decreased binding at PND 14 in striatum and nucleus accumbens after MAO-A or combined MAO-AB administration. More detailed evaluation of this effect would require further investigation. The relative overall lack of an MAO effect on DAT maybe due to the primary deamination of dopamine through catechol-O-methyl transferase pathway [55,56,57]. 

The present studies used the same protocol for developmental inhibition of MAO activity as that reported previously [26]. In that study, radiochemical analysis of mouse brain showed that treatment with clorgyline (MAO-A group) reduced MAO-A activity by 25% and MAO-B activity by 29%, while deprenyl treatment (MAO-B group) was less effective (MAO-A: −15%, MAO-B: −28%). The experimental group treated with both clorgyline and deprenyl showed the highest inhibition of both enzymes, with 41% and 42% inhibition for MAO-A and MAO-B, respectively [26]. The present findings suggest that the critical level of MAO inhibition, which subserves SERT reduction, is in 25–40% range. 

There are conflicting reports regarding the ontogeny of SERT binding. Tarazi et al. [38] examined SERT densities in the striatum of rats at 7 developmental time-points ranging from PND 7–60 and found that SERT increases steadily throughout development. However, Galineau et al. [37] describe a tri-phasic pattern of development in rats where SERT binding is maximal between PND 0–14, receding between PND 14–28, followed by a plateau in binding through adulthood. Control samples drawn from the present study suggest that the SERT ontogeny in mice best resembles the developmental profile described by Tarazi et al. [38]. SERT densities in the raphe are near adult levels by PND 14, and in all other areas examined SERT densities increase through to adulthood. 

Our previous work showed that perinatal MAO inhibition increased aggressive behaviors, most noticeable in MAO-A/B treated subjects [26]. Neurologically, pups used in the present study, like those reported by Mejia et al. [26], were physically unaffected by treatment regime. Likewise, motor activity was not different between experimental groups in either the current or previous study [26]. There were also no differences between groups with respect to the behavioral measurements of stereotypies and exploratory behaviour. During development, serotonin displays both positive and negative autoregulation of neuronal terminal density, particularly in the cortex [54,58]. 

Although neither dopamine nor serotonin were measured directly in this study, the inhibition of MAO-A, especially during early embryogenesis, can be inferred to increase brain levels of serotonin. Furthermore, since DAT levels were largely unaffected, the alterations seen in SERT levels reinforces the idea that the changes of SERT are not specifically the consequences of MAOA/B blockade, but rather a potential alteration in serotonin autoregulation, which occurs through both direct and indirect pathways. The indirect pathway involves the astroglial-derived S-ß100 protein, which is released following serotonin stimulation of the 5-HT_1A_ receptor [59,60]. S-ß100 then increases outgrowth of neurites on serotonin neurons [61,62]. Although physiological concentrations of serotonin promote synaptogenesis, Chubakov et al. [63] found that both under- and over-stimulation during rat embryonic development (E12-17) decreased 5-HT_1A_ receptor densities [64,65]. In the direct pathway, growth is inhibited by excess serotonin [66,67] stimulation of the 5-HT_1B_ autoreceptor [54].

Elevated levels of serotonin during embryogenesis could thus decrease the density of 5-HT_1A_ receptors [64,65], reducing target cell differentiation [64,68,69]. Increased concentrations of serotonin during development would also exacerbate negative autoregulation of the serotonin neuronal terminal field, particularly in the cortex [54]. This decreased innervation of the cortex would contribute to the impulsive and aggressive behavioral responses previously reported for this model [26]. 

With an estimated 8% of women using antidepressants that raise serotonin levels during pregnancy [70,71], the association between elevated serotonin levels in utero and the risk for altered neurodevelopment remains an important, albeit controversial topic [43,44,45,46,47,72,73,74]. The use of serotonin specific reuptake inhibitors (SSRIs) has been weakly associated with delayed motor development [75] and elevated corticotropin-releasing hormone levels [76] and has been proposed to be a risk for autism spectrum disorder [77,78,79,80]. Although a causative relationship between the clinical use of SSRIs during pregnancy and altered neurodevelopment has not been established, its use should be balanced against the maternal benefits of antidepressants. 

## 5. Conclusions

The major finding reported here is that the prenatal inhibition of MAO-A and B significantly and specifically reduced SERT binding by up to 25% in the cortex and raphe nucleus at defined developmental stages, persisting into adulthood. This pattern of low SERT binding is consistent with the behavioral changes previously described in this model [26]. The data suggest that inhibition of MAO activity by 25–40% effectively alters serotonin innervation.

The developmental sensitivity to fluctuating levels of serotonin raises important issues regarding the ingestion of psychoactive drugs during pregnancy. The current study not only warns about the long-term effects of the use of certain antidepressants during pregnancy, but also of other drugs such as cocaine, nicotine, and ethanol which inhibit MAO or increase extracellular levels of serotonin [81,82,83,84]. 

## Figures and Tables

**Table 1:** Motor Activity at PND 35 in MAO-inhibited and Control Mice.

Group	Locomotor Activity	Stereotypy Count	Margin Time	Center Time
**A**	1626 ± 135 cm	20.0 ± 2.1	157.3 ± 4.1 s	21.7 ± 4.1 s
**B**	1732 ± 173 cm	22.2 ± 2.4	150.2 ± 3.7 s	28.8 ± 3.7 s
**AB**	1571 ± 156 cm	18.3 ± 2.3	153.2 ± 4.1 s	25.8 ± 4.1 s
**C**	1970 ± 198 cm	15.5 ± 2.2	151.3 ± 3.2 s	27.7 ± 3.2 s

Behavioral measures (locomotor activity, incidence of stereotypies, performance in an open field test) were obtained as described in detail in Methods. Neither average locomotor activity (F_3,20_ = 1.12, *p* = 0.37) nor number of stereotypies (F_3,20_ = 1.55, *p* = 0.23) differed between experimental groups. Time spent along the sides (margin time; F_3,20_ = 0.68, *p* = 0.57) or in the center of the open field also did not vary across groups (F_3,20_ = 0.681, *p* = 0.57). Data are presented as mean ± standard error. A, monoamine oxidase (MAO) A enzyme-inhibited mice (*n* = 6); B, MAO-B enzyme-inhibited mice (*n* = 6); AB, MAO-A and MAO-B enzyme-inhibited mice (*n* = 6); C, control animals (*n* = 6).

**Table 2:** Control Levels of Serotonin and Dopamine Transporters during Postnatal Development.

		**Serotonin Transporter, fmol/mg Tissue**						
PND	Striatum	Hippocampus		Cortex		Raphe		Sub nigra
14	0.39 ± 0.07	0.42 ± 0.19		0.42 ± 0.14		1.54 ± 0.31		1.47 ± 0.23
35	1.03 ± 0.16	0.89 ± 0.15		0.73 ± 0.12		1.57 ± 0.18		2.31 ± 0.35
90	1.10 ± 0.10	0.95 ± 0.09		0.84 ± 0.09		1.7 ± 0.37		2.07 ± 0.18
	**Dopamine Transporter, fmol/mg Tissue**								
PND	Nuc accumbens		Striatum		Loc coeruleus		Sub nigra		
14	1.29 ± 0.31		1.35 ± 0.18		0.91 ± 0.13		0.8 ± 0.09		
35	1.30 ± 0.07		1.39 ± 0.06		0.75 ± 0.2		0.71 ± 0.06		
90	1.64 ± 0.16		1.66 ± 0.13		0.61 ± 0.02		0.83 ± 0.15		

Densities of SERT and DAT (expressed as fmol/mg tissue) were measured by radioreceptor binding, as described in detail in Methods. In control animals, SERT density increased significantly between postnatal days 14 and 90 in cortex (F_2,13_ = 18.87, *p* = 0.0001), hippocampus (F_2,13_ = 19.58, *p* = 0.0001), striatum (F_2,14_ = 60.93, *p* < 0.0001) and substantia nigra (F_2,14_ = 14.05, *p* = 0.0004). Raphe levels of SERT did not increase significantly between days 14 and 90. There were no significant changes in DAT levels between 14 and 90 days post-natal.

**Table 3:** Regional SERT and DAT levels.

	Serotonin Transporter (fmol/mg Tissue)					Dopamine Transporter (fmol/mg Tissue)		
	Striatum	Hippocampus	Cortex	Raphe	Sub Nigra	Nuc. Accumbens	Striatum	Sub Nigra
**PND 14**								
C	0.39 ± 0.07	0.42 ± 0.19	0.42 ± 0.14	1.54 ± 0.31	1.47 ± 0.23	1.29 ± 0.31	1.35 ± 0.18	0.80 ± 0.09
A	0.44 ± 0.11	0.51 ± 0.10	0.44 ± 0.04	1.33 ± 0.32	1.22 ± 0.23	0.96 ± 0.30	1.17 ± 0.15	0.60 ± 0.08
B	0.40 ± 0.12	0.50 ± 0.09	0.45 ± 0.09	1.15 ± 0.16	1.23 ± 0.14	1.11 ± 0.30	1.38 ± 0.41	0.82 ± 0.19
AB	0.39 ± 0.18	0.49 ± 0.12	0.38 ± 0.08	1.11 ± 0.24	1.29 ± 0.34	0.84 ± 0.21	1.11 ± 0.14	0.60 ± 0.12
**PND 35**								
C	1.03 ± 0.16	0.89 ± 0.15	0.73 ± 0.12	1.57 ± 0.18	2.31 ± 0.35	1.30 ± 0.07	1.39 ± 0.06	0.71 ± 0.06
A	1.01 ± 0.19	0.84 ± 0.22	0.73 ± 0.15	1.52 ± 0.26	2.21 ± 0.32	1.71 ± 0.59	1.70 ± 0.70	0.73 ± 0.31
B	0.98 ± 0.13	0.79 ± 0.16	0.70 ± 0.09	1.60 ± 0.21	2.29 ± 0.28	1.69 ± 0.53	1.71 ± 0.53	0.75 ± 0.28
AB	0.89 ± 0.13	0.89 ± 0.26	0.59 ± 0.21	1.27 ± 0.46	2.04 ± 0.40	1.71 ± 1.03	1.64 ± 0.74	0.72 ± 0.33
**PND 90**								
C	1.10 ± 0.10	0.95 ± 0.09	0.84 ± 0.09	1.7 ± 0.37	2.07 ± 0.18	1.64 ± 0.16	1.66 ± 0.13	0.83 ± 0.15
A	0.97 ± 0.15	0.79 ± 0.09	0.67 ± 0.10	1.52 ± 0.26	1.96 ± 0.14	1.46 ± 0.29	1.59 ± 0.33	0.76 ± 0.26
B	0.99 ± 0.15	0.90 ± 0.09	0.72 ± 0.08	1.33 ± 0.11	2.03 ± 0.06	1.73 ± 0.19	1.85 ± 0.35	0.86 ± 0.32
AB	0.90 ± 0.09	0.89 ± 0.14	0.63 ± 0.12	1.23 ± 0.28	1.96 ± 0.14	1.59 ± 0.28	1.75 ± 0.33	0.77 ± 0.27

Densities of SERT and DAT (fmol/mg tissue) were measured across different regions as describe in the methods. There was main effect of MAO inhibition on SERT binding in the raphe (F_3,51_ = 6.07, *p* = 0.0015; AB versus C, *p* < 0.0001; B versus C, *p* < 0.01; and AB versus A, *p* < 0.01) and cortex (F_3,52_ = 3.59, *p* = 0.02; AB versus C, *p* = 0.0004; AB versus B, *p* < 0.05; and AB versus A, *p* < 0.02). There were no significant effects of treatment on SERT binding in the hippocampus, striatum or substania nigra or on DAT expression in the striatum, nucleus accumbens or substantia nigra.
